# A novel maxillary transverse deficiency diagnostic method based on ideal teeth position

**DOI:** 10.1186/s12903-023-02790-w

**Published:** 2023-02-07

**Authors:** Ting Ma, Yan-hai Wang, Chun-xi Zhang, Dong-xu Liu

**Affiliations:** 1 Department of Orthodontics, School and Hospital of Stomatology, Cheeloo College of Medicine, Shandong University and Shandong Key Laboratory of Oral Tissue Regeneration and Shandong Engineering Laboratory for Dental Materials and Oral Tissue Regeneration and Shandong Provincial Clinical Research Center for Oral Diseases, No.44-1 Wenhua Road West, Jinan, 250012 Shandong China; 2 Jinan Nursing Vocational College, No. 3636 Gangxi Road, Licheng District, Jinan, 250000 Shandong China; 3 Center of Oral Medicine, Qingdao Municipal Hospital, No. 1 Jiaozhou Road, Qingdao, 266011 Shandong China

**Keywords:** Ideal teeth positions, Maxillary transverse deficiency, Mild skeletal Class III malocclusion, Skeletal Class I malocclusion, Digital align technology

## Abstract

**Background:**

This study proposed a novel maxillary transverse deficiency diagnostic method and evaluated the skeletal Class I and the mild skeletal Class III groups.

**Methods:**

Pre-treatment data from 30 mild skeletal Class III and 30 skeletal Class I patients were collected and uploaded to the Emeiqi Case Management System to design the ideal teeth positions. On these positions, the first bi-molars width was measured at the central fossa and center resistance, the maxillary first bi-premolars width was measured at the central fossa, and the mandibular first bi-premolars width was measured at the distal contact point by Mimics, then width differences of two groups were calculated respectively.

**Results:**

At ideal teeth positions, there was no statistically significant difference in the maxillomandibular width in the premolar area between the two groups, but there was in the molar area, and this difference was caused by the difference in mandible width between the two groups.

**Conclusions:**

We proposed a new transverse diagnostic method and found that even the Class I group was not quite up to standard in the molar area on ideal teeth positions, and the Class III group had more severe maxillary transverse deficiency than the Class I group. Meanwhile, the maxillary transverse deficiency in the Class III group was mainly caused by the larger width of the mandible.

## Introduction

Maxillary transverse deficiency (MTD) is a skeletal deficiency of the upper jaw that was first proposed by Angell E [1]. The condition is pervasive in orthodontic patients [2, 3] yet more likely to be overlooked than sagittal and vertical discrepancies [4]. Such missed cases are especially likely for mild skeletal Class III patients, who have teeth compensation and no clinical crossbite in the posterior area [5]. Due to the transverse dimension being the earliest to establish, it can adversely affect the consequent sagittal and vertical growth. This alteration in development occurs in the presence of obvious initial transverse discrepancies such as facial asymmetry, posterior crossbite, and scissor bite, particularly following a lack of timely correction [3, 6, 7]. Therefore, choosing a reliable and valid diagnostic method for transverse abnormalities is crucial before planning patients’ treatment.

Recently, increasingly more research has focused on the diagnosis of MTD, facets including clinical evaluation [8, 9], model analysis [10–12], and radiographic measurements, which had been recommended for assessment of the condition [13]. Among these methods, Andrews’ Element III Analysis defined the optimal jaw widths [14] and is the most widely used model analysis method at present. Posteroanterior cephalograms (PACs) were once considered the most available and reliable methods, however, the conventional two-dimensional (2D) images of skeletal structures have technical limitations that reduce the accuracy of landmark location [15, 16]. Cone-beam computed tomography (CBCT) shows more invariability and reproducibility for transverse measurements [17], such examples include the University of Pennsylvania Cone-Beam CT Analysis [18], the Yonsei transverse index [19], and Case Western University’s (CWRU) transverse analysis [20] et al.

Nevertheless, all these methods are based on the pre-treatment teeth positions instead of the final teeth positions. When we assess a patient's condition, the ideal final position of the dentition should be used as a criterion, which follows the "start with the end". Besides, orthodontic and orthopedic appliances allow teeth to move within the alveolar bone or to move with the jaws in three dimensions, these position changes of the teeth and jaw in three dimensions can affect the relative transverse relationship [21]. Therefore, We propose a goal-oriented, inverse approach to assess maxillary width: Basing on The Six Elements of Orofacial Harmony [22] (except element III), the ideal maxillary and mandibular dentition and tooth positions were obtained separately using digital alignment techniques, and the coordination of maxillary and mandibular transverse widths was evaluated in this positions.

## Subjects and methods

### Subjects

This study was accepted by the Research Ethics Board of School of Stomatology, Shandong University (Protocol NO.20211212). Among the outpatients who visited the Department of Orthodontics of the School of Stomatology, Shandong University from 2018 to 2022, 30 skeletal Class I patients and 30 mild skeletal Class III patients were included in this study.

The inclusion criteria were as follows: (1) all permanent teeth, including the second molars, were present and fully erupted to the occlusal plane, (2) having a non-extraction comprehensive orthodontic treatment plan, (3) crowding of less than 4 mm existed in each arch, (4) that the mild skeletal Class III group should meet the mesial relationship of bilateral molars in centric position, ANB angle was less than 0.7, and no crossbite existed in the posterior teeth, (5) that the skeletal Class I group should have an ANB angle between 0.7° and 4.7°. The exclusion criteria included: (1) severe abnormal morphology, restoration, fracture of first permanent molars root, (2) periodontal diseases, and (3) history of previous orthodontic or orthopedic treatment, trauma, or surgery in the oral and maxillofacial region.

The pre-treatment CBCT images (NewTom 5G; NewTom, Verona, Italy; 0.3-mm voxel size; parameter: 110 kV, 5 mA), intraoral scan data (iTero Element II; Align Technologies, Shanghai, China), panoramic radiographs, lateral cephalometric radiographs, extraoral photographs, and intraoral photographs were retrospectively selected from the past orthodontic records. They were not specifically taken for this research but orthodontic treatment.

### Study design

**Fig. 1:**
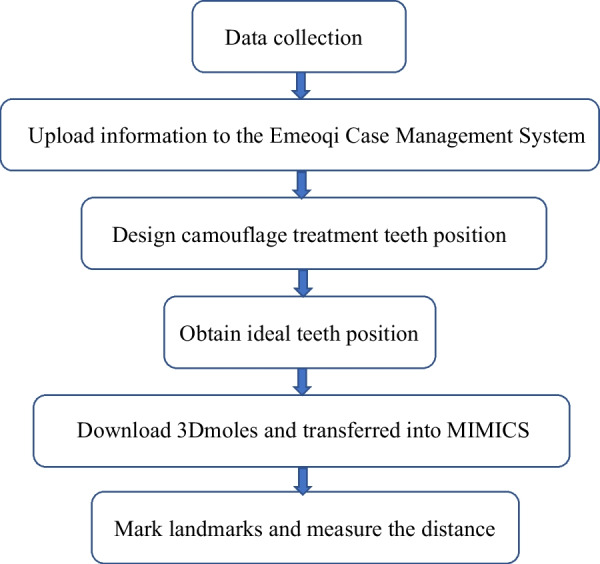
Schematic diagram of the experimental process

**Fig. 2:**
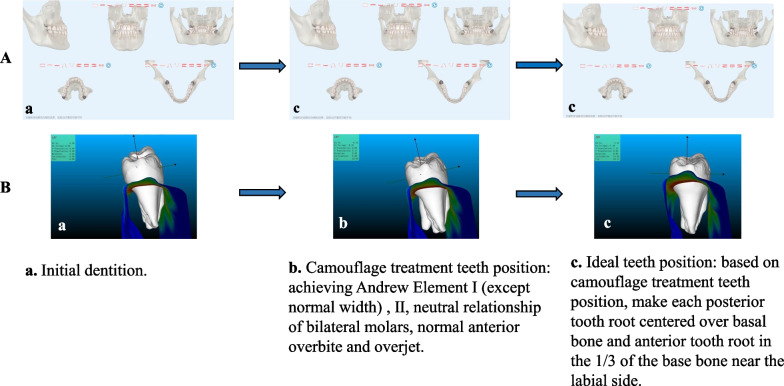
The process of getting the Ideal teeth position. **A** An Ideal teeth position's design process. **B** Schematic diagram torque variation of mandibular first molars at different teeth positions: a. Initial dentition. b. Camouflage treatment teeth position. c. Ideal teeth position

As Fig. 1. showed, firstly, subject data were uploaded to the online Emeiqi Case Management System (Hangzhou Meiqi Technology Co., Ltd., China, https://doctor.i-align.com/) (which is a patented product of Meiqi Technology Co., Ltd., an invisible orthodontic company with professional engineers) to design camouflage orthodontic treatment teeth positions and ideal teeth positions. This was done in the following manner:

① Using the MQStudio Root Bone Analysis Tool (Hangzhou Meiqi Technology Co., Ltd., China) (which also is a patented product of Meiqi Technology Co., Ltd.) to extract the root and alveolar bone data through CBCT and build the corresponding model, convert it into the surface mesh form, and convert the oral scan data into the same form, and then perform the two data on this modality. After registration and output, a 3D model containing accurate crown, root, and alveolar bone is obtained.
② Engineers aligned and leveled teeth according to the treatment targets that doctor designed individually for each patient, including the achievement of (a) Andrews’ Element I (the contact areas abut; each crown inclined so that its occlusal surface can interface and function optimally with the teeth in the opposing arch; the curve of Spee depth falling between 0 and 2.5 mm; the core line length equalling the sum of the mesiodistal diameters of the teeth in the arch, (b) Andrews’ Element II (the optimal anteroposterior jaw positioning: based on (a), the Facial Axis points (FA pts.) of maxillary central incisors are on the GALL, while in centric relation, the mandibular central incisors are coupled with the maxillary incisors in an optimal maxilla) [14], (c) the anterior overbite and overjet being classed as normal, and (d) a neutral relationship of bilateral molars in centric position. Technicians then imported the alveolar bone model, analyzed the root-bone relationship, and further adjusted the occlusion to ensure masticatory function and improve the facial appearance. At this time, the teeth positioning for camouflage treatment was obtained.
③ Further, root torque or positive axis movement of the teeth was performed to remove the compensation of the lingual or buccal torque of the maxillary and mandibular teeth, and the parallelism of the roots was consistent (Fig. 2).
④ Analyzing the relative positions of the teeth roots and the alveolar bone and confirming that the positions of the teeth roots in the alveolar bones met the requirements of Andrews’ Elements I and II. Then, the ideal teeth positions were obtained.

### 3D measurement

**Fig. 3:**
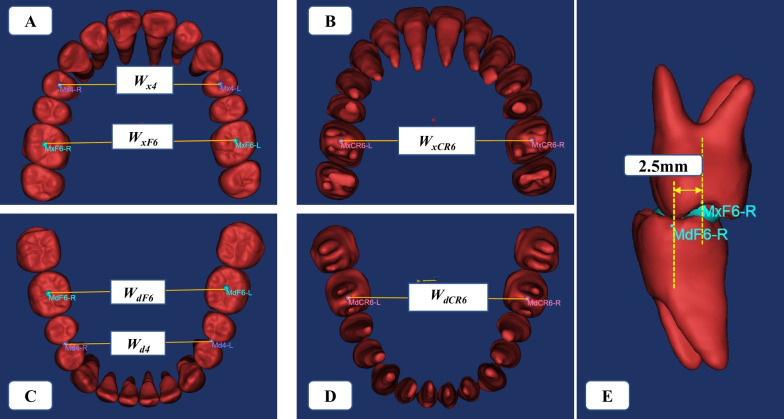
The dental landmarks and measurements on maxilla and mandible. **A**, **B** The maxillary landmarks and measurements. **C**, **D** The mandibular landmarks and measurements. **E** The ideal width between maxilla and mandible in one side is 2.5 mm

The teeth and jaws 3D models at the ideal teeth positions were downloaded in Stereo Lithography (STL) formats and transferred into Materialise’s Interactive Medical Image Control System (MIMICS, version 21.0; Leuven, Belgium) software package to measure. All 3D models were coded and randomized to blind the investigator who took the measurements. The dental landmarks, relative evaluation measurements, and their definitions are shown in Fig. 3 and Table 1.

**Table 1:** The dental landmarks, relative evaluation measurements and their definitions

Dental Landmarks/measurements	Definitions
*Maxilla (x)*	
MxF6-L/ MxF6-R	The central fossa of maxillary left/right first molar at ideal teeth position
MxCR6-L/ MxCR6-R	The center resistance of maxillary left/right first molar at ideal teeth position
Mx4-L/ Mx4-R	The central fossa of maxillary left/right first premolar at ideal teeth position
W_xF6_	Width between MxF6-L and MxF6-R
W_xCR6_	Width between MxCR6-L and MxCR6-R
W_x4_	Width between Mx4-L and Mx4-R
*Mandible (d)*	
MdF6-L/ MdF6-R	The central fossa of the mandibular left/right first molar at ideal teeth position
MdCR6-L/ MdCR6-R	The center resistance of the mandibularl left/right first molar at ideal teeth position
Md4-L/ Md4-R	The distal contact point of the mandibular left/right first premolar at ideal teeth position
W_dF6_	Width between MdF6-L and MdF6-R
W_dCR6_	Width between MdCR6-L and MdCR6-R
W_d4_	Width between Md4-L and Md4-R
*Difference (x measurement – d measurement)*	
D_F6_	Difference between W_xF6_ and W_dF6_
D_CR6_	Difference between W_xCR6_ and W_dCR6_
D_4_	Difference between W_x4_ and W_d4_

### Statistics

Based on prior power analyses and using G.Power software (Erdfelder, Faul, & Buchner, German) to determine the sample sizes, with an alpha of 0.05, and a power of 0.85, the number of subjects should be 30 for each group. Using Statistical Package for the Social Sciences (SPSS, version 26.0; IBM, Armonk, NY) to perform statistical analysis. First, All the measurements were carried out by a trained and calibrated investigator independently at a 2-week interval, and all values were measured on the same computer to prevent performance bias [23]. Second, the intraclass correlation coefficients (ICC; 2-way random, absolute agreement, Single measurements) which evaluated and reflected the intra-group reliability of the measurements, and its classification definition is as follows: excellent (> 0.9), good (0.75–0.9), moderate (0.5–0.75), or poor (< 0.5). Third, the Shapiro–Wilk test was used to verify the normality of data distribution. Except for values of ***D***_***F6***_ in different sex groups, the rest of the results conformed to a normal distribution. The Independent *t*-test was applied for comparison data that conformed to a normal distribution, values were presented as mean (SD); the Mann–Whitney* U*-test was applied for comparison data that did not conform to a normal distribution, and values were presented as median (IQR). A *P* value less than 0.05 indicated statistical significance.

## Results

A total of 60 patients were randomly enrolled in this study, including 30 skeletal Class III and 30 skeletal Class I subjects respectively, the skeletal Class III group included 14 male patients and 16 female patients, with a mean (SD) age of 17.9 (0.9) years and the skeletal Class I group including 9 male patients and 23 female patients, with a mean (SD) age of 21.4 (1.1) years. The *ICC* with 95% confidence intervals for intraexaminer reliability were all above 0.85, indicating good to excellent reliability. The results showed no significant sex differences between the two groups in all measurements (Table 2), which meant errors caused by gender differences were minimal in our study.

**Table 2:** The measurement results of skeletal Class I and mild skeletal Class III (unit: mm)

Group		D_F6_	D_CR6_	D_4_
Skeletal Class I	Man	2.90 ± 1.67	1.57 ± 2.46	1.64 ± 1.61
	Female	3.20 ± 1.79	1.05 ± 1.97	1.49 ± 1.64
	*P*-values	0.665^a^	0.544^a^	0.827^a^
	ICC	0.937	0.940	0.986
	95% CI	0.827–0.978	0.830–0.980	0.961–0.965
Mild skeletal Class III	Man	1.79 ± 2.40	− 0.79 ± 2.10	1.43 ± 1.29
	Female	2.41 ± 3.22	− 1.78 ± 2.79	0.86 ± 1.37
	*P*-values	0.618^b^	0.289^a^	0.250^a^
	ICC	0.877	0.957	0.872
	95% CI	0.173–0.970	0.876–0.985	0.660–0.955

D_F6,_ Difference between maxilla and mandible width measuring on first intermolar fossa. D_CR6_, Difference between maxilla and mandible width measuring on first intermolar center resistance. D_4_, Difference between maxilla and mandible width measuring on interpremolar landmarks

^a^*P*-values were calculated using independent *t*-test, values are presented as mean ± SD

^b^*P*-values were calculated using Mann–Whitney U-test, values are presented as median ± IQR

Table 3. showed the measurement and statistical results in two groups. All measurement results conformed to normal distributions. The independent *t*-test showed that there were no significant statistical intergroup differences in *W*_*xF6*_ (*P* = 0.795),* W*_*xCR6*_ (*P* = 0.187), *W*_*x4*_ (*P* = 0.795), *W*_*d4*_ (*P* = 0.480),* D*_*4*_ (*P* = 0.285). However, *W*_*dF6*_ of the Class III group and Class I group was 47.72 ± 1.97 mm and 46.44 ± 2.32 mm respectively, which showed significant statistical differences (*P* = 0.025); *W*_*dCR6*_ of the Class III group and Class I group was 49.33 ± 1.97 mm and 47.60 ± 2.17 mm respectively, which showed significant statistical difference (*P* = 0.002); *D*_*F6*_ of Class III group and Class I group was 1.97 ± 2.15 mm and 3.11 ± 1.73 mm respectively, which showed significant statistical difference (*P* = 0.023); *D*_*CR6*_ of Class III group and Class I group was − 1.32 ± 2.50 mm and 1.21 ± 2.10 mm respectively, which also showed significant statistical difference (*P* = 0.000).

**Table 3:** The measurement results of skeletal Class I and mild skeletal Class III (unit: mm)

Variables	Skeletal Class I	Mild skeletal Class III	*P*-value
	Mean ± SD	Mean ± SD	
*Maxilla (x) measurement*			
W_xF6_	49.55 ± 1.20	49.70 ± 2.24	0.795
W_xCR6_	48.81 ± 2.27	48.01 ± 2.42	0.187
W_x4_	39.94 ± 1.64	39.84 ± 1.54	0.795
*Mandible (d) measurement*			
W_dF6_	46.44 ± 2.32	47.72 ± 1.97	0.025*
W_dCR6_	47.60 ± 2.17	49.33 ± 1.97	0.002**
W_d4_	38.41 ± 2.16	38.75 ± 1.44	0.480
*Difference (x measurement – d measurement)*			
D_F6_	3.11 ± 1.73	1.97 ± 2.15	0.028*
D_CR6_	1.21 ± 2.10	− 1.32 ± 2.50	0.000***
D_4_	1.54 ± 1.61	1.12 ± 1.34	0.285

W_xF6_/W_dF6_, Width of first intermolar central fossa. W_xCR6_ / W_dCR6_, Width of first intermolar center resistance. W_x4_ / W_d4_, Width of maxillary first interpremolar central fossa or mandibular first interpremolar distal contact point. D_F6_, Difference between W_xF6_ and W_dF6_. D_CR6_, Difference between W_xCR6_ and W_dCR6_. D_4_, Difference between W_x4_ and W_d4_

*P-values* were calculated using independent *t*-test

*P < 0.05; **P < 0.01; ***P < 0.001

## Discussion

Orthodontic treatment should abide by the concept of ‘starting with the end’, and the same should be true of orthodontic diagnostic methods. Clinicians may easily underestimate transverse discrepancies that are masked by teeth compensation. Therefore, in this study, we chose skeletal Class I and mild skeletal Class III subjects without clinically posterior crossbites to aligned the teeth at the ideal teeth positions, evaluated and compared their transverse widths, our study was dedicated to the diagnostic effectiveness of MTD. In our study, we chose the fossa of the maxillary first molar as the landmark at the crown instead of the mesiopalatal cusp, which corresponds to the fossa of the mandibular first molar, because the fossa was more stable and reproducible than the cusp, which may have attrition or abrasion. We set the standard of maxillomandibular width difference at first bi-molar central fosses to 5 mm, because research has shown that in instances where the mesiopalatal cusp of the maxillary first molar and the mesiobuccal cusp of the mandibular first molar are centered in the central fossae of the molars in the opposing arch, the distance between the cusp tips will be approximately 2.5 mm (for a total amount of 5 mm) [18, 24, 25] (Fig. 3E).

The present study found that the D_F6_ and D_CR6_ were both significantly lesser in the mild skeletal Class III group than in the skeletal Class I group and the D_F6_ of the former was markedly less than 5 mm, it indicated that the mild skeletal Class III group indeed had maxillary transverse deficiencies, this finding was consistent with previous studies measuring transverse widths at pre-treatment dentations [19, 26, 27]. However, the mean of the first bi-molar width differences in skeletal Class I was also lesser than 5 mm, suggesting that even skeletal Class I patients may possess a certain degree of maxillary transverse deficiency, on the one hand, this is due to the fact that most patients who come to the hospital still have a certain degree of deformity even for skeletal Class I malocclusion, on the other hand, the skeletal classification is carried out according to the sagittal direction, which cannot represent normal development in the transverse direction. This particular finding deviated from the previous perception and hence requires further research to confirm it. Most previous research defaulted that the values of skeletal Class I patients could represent the normal standards, but malocclusions happen in three dimensions and skeletal classification only reflects the sagittal conditions. Thus, this finding prompted us not to ignore transverse deficiency evaluation in skeletal Class I patients, and for those diagnostic methods that used skeletal Class I values as the standard criteria [19], we should use them with caution, or else in combination with other diagnostic methods. In addition, in clinical practice, 3 mm is typically chosen as the standard to judge whether the patient needs arch expansion treatment, if the width of the mandibular base bone exceeds 3 mm than the maxillary base bone, bone expansion treatment must be considered [6, 10, 28]. Clinically, skeletal class I patients usually do not require additional arch expansion devices, which, combined with the results of this study, confirms this "3 mm critical value" point of view.

There was no statistical difference between W_xF6_ and W_xCR6_, the difference was mainly between W_dF6_ and W_dCR6_. This finding indicated the MTD of the skeletal Class III group mainly caused by mandibular prognathism, it was consistent with previous research [27], Spalj et al. also found that mandibular prognathism with a normal maxilla is the most common differential skeletal type in skeletal Class III patients [29]. But Hwang et al. considered that there was no significant difference in the mandibular transverse width at the alveolar crest or mid root level between the Class III and Class I groups, and the maxillary buccolingual alveolar width at the mid root level was significantly smaller in the Class III group compared with that of the Class I group [7]. So larger sample sizes and more detailed taxonomy studies are required to confirm the conclusion, and it is worth noting that the study of Hwang et al. measured at the alveolar crest, so the alveolar thickness may have influenced the results, alongside a possible impact caused by subjects’ difference, as our study only included mild skeletal Class III patients rather than all skeletal Class III patients.

Besides, the first bi-molar width differences measured at center resistance in the two groups in our study were larger than Koo’s results, this difference may be due to ethnographic differences or sample selection, but it is also likely that even if the center resistance is more stable than the other positions of the tooth, due to its’ position changes with the tooth three-dimensional movement during orthodontic treatment, evaluating transverse width from the center resistance at pre-treatment dentitions is still inaccurate and inadequate.

The first bi-premolar widths showed no statistically significant intergroup differences. This contrasted with previous research findings [19] which showed remarkable differences in basal arch widths between the normal occlusion versus the Class III malocclusion groups in the first premolar area. Such differences might be due to the premolar teeth compensation in Class III malocclusion and change in dental arch shape during teeth alignment. Besides, we found that there were no significant gender differences in all transverse width differences between the groups, but many studies proved gender differences in the widths of the maxilla or mandible [7, 26, 29]. It indicated that it was more reliable and convenient to choose maxillomandibular width difference in the transverse width evaluation instead of the single jaw width.

Jacobs et al. [21] proposed that maxillary transverse deficiencies should be corrected and reassessed after correcting the sagittal relationship. The Six Elements of Orofacial Harmony defined that the maxillomandibular transverse measurement should be taken on the optimal arch. In this study, thanks to digital align technology, we could simulate the ideal teeth positions that draw on the principles advocated by Jacobs and The Six Elements of Orofacial Harmony, so the results can more realistically reflect the maxillomandibular transverse width. McNamara et al. and Moyers [30, 31] reported that the maxillary intermolar width was established at age 12 for girls and increased by 1.4 mm between the ages of 12 to 18 for boys. Therefore, our study’s results reflected permanent dentition characteristics.

As Wagner and Chung noted that there was a relationship between transverse growth and vertical facial types [32], so further studies are needed to investigate the characteristics of other dentitions and different vertical bone profiles. This method provided a new diagnostic perspective, and with the future development of intelligent teeth arrangement software alongside larger sample sizes, this method could become both more scientific and convenient and help to explore the gold standards of transverse width difference.

## Conclusions

Since the orthodontic treatment process involves six degrees of freedom in three-dimensional space and patients who come to seek treatment have certain abnormalities, the purpose of orthodontics is to correct or improve these abnormalities through the movement of teeth or jaws. This means that the orthodontic landmarks used in the diagnostic method based on pre-treatment dentition will move both during and after treatment, hence they are inaccurate and insufficient in evaluating maxillary transverse deficiency. We proposed a new method that simulated the ideal tooth position by using digital align technology. This position is not only close to the optimal treatment goals but also close to the after-treatment tooth position, which can better reflect the existence of maxillary transverse deficiency. Using this method, we measured skeletal Class I and mild skeletal Class III patients to find that:

1. The Class III group had more severe maxillary transverse deficiency than the Class I group in the molar area, but the Class I group also failed to meet the standard.
2. Compared with the Class I group, the maxillomandibular width difference in the Class III group was mainly caused by mandibular prognathism.
3. Even if the center resistance was more stable than other positions of the tooth, due to the teeth movement, there was still an error in the transverse measurement from the center resistance at pre-treatment dentitions.

## Data Availability

The datasets used and/or analyzed during the current study are available from the corresponding author on reasonable request.

## Abbreviations

CBCT: Cone-beam computed tomographye
Mimics: Materialse's interactive medical image control system
MTD: Maxillary transverse deficiency
DICOM: Digital imaging and communications in medicine
STL: Stereolithography

## References

[1] Angell D (1860). Treatment of irregularity of the permanent or adult teeth. Dent Cosmos.

[2] Musich D, Busch MJ (2007). Early orthodontic treatment: current clinical perspectives. Alpha Omegan.

[3] Lee KJ, Choi SH, Choi TH, Shi KK, Keum BT (2018). Maxillary transverse expansion in adults: Rationale, appliance design, and treatment outcomes. Semin Orthodontics.

[4] Tamburrino RK, Shah SR, Fishel DLW (2014). Periodontal rationale for transverse skeletal normalization. Orthodontic Pract.

[5] Podesser B, Williams S, Bantleon HP, Imhof H (2004). Quantitation of transverse maxillary dimensions using computed tomography: a methodological and reproducibility study. Eur J Orthodont.

[6] Proffit WR, Fields HW, Larson B, Sarver DM (2018). Contemporary orthodontics.

[7] Hwang S, Song J, Lee J, Choi YJ, Chung CJ, Kim KH (2018). Three-dimensional evaluation of dentofacial transverse widths in adults with different sagittal facial patterns. Am J Orthod Dentofacial Orthop.

[8] McNamara JA (1981). Influence of respiratory pattern on craniofacial growth. Angle Orthod.

[9] Betts NJ, Vanarsdall RL, Barber HD, Higgins-Barber K, Fonseca RJ (1995). Diagnosis and treatment of transverse maxillary deficiency. Int J Adult Orthodon Orthognath Surg.

[10] Nimkarn Y, Miles PG, O'Reilly MT, Weyant RJ (1995). The validity of maxillary expansion indices. Angle Orthod.

[11] Joondeph DR, Riedel RA, Moore AW (1970). Pont's index: a clinical evaluation*. Angle Orthod.

[12] Dalidjan M, Sampson W, Townsend G (1995). Prediction of dental arch development: an assessment of Pont's Index in three human populations. Am J Orthod Dentofacial Orthop.

[13] Lokesh Suri PT (2008). Surgically assisted rapid palatal expansion: a literature review. Am J Orthod Dentofacial Orthop.

[14] Andrews LF, Andrews WA. The six elements of orofacial harmony. Andrews J. 2000.

[15] Malkoc S, Sari Z, Usumez S, Koyuturk AE (2005). The effect of head rotation on cephalometric radiographs. Eur J Orthod.

[16] Sawchuk D, Currie K, Vich ML, Palomo JM, Flores-Mir C (2016). Diagnostic methods for assessing maxillary skeletal and dental transverse deficiencies: a systematic review. Korean J Orthod.

[17] Tai B, Goonewardene MS, Murray K, Koong B, Islam SM (2014). The reliability of using postero-anterior cephalometry and cone-beam CT to determine transverse dimensions in clinical practice. Aust Orthod J.

[18] Tamburrino RK, Boucher NS, Vanarsdall RL, Secchi A (2010). The transverse dimension-diagnosis and relevance to functional occlusion. RWISO J.

[19] Koo YJ, Choi SH, Keum BT, Yu HS, Hwang CJ, Melsen B, Lee KJ (2017). Maxillomandibular arch width differences at estimated centers of resistance: comparison between normal occlusion and skeletal Class III malocclusion. Korean J Orthodontics..

[20] Nervina JM, Kapila SD, Flores-Mir C, Kapila SD (2014). Assessment of maxillary transverse deficiency and treatment outcomes by cone beam computed tomography. Cone beam computed tomography in orthodontics: indications, Insights, and Innovations.

[21] Jacobs JD, Bell WH, Williams CE, Kennedy JWIII (1980). Control of the transverse dimension with surgery and orthodontics. Am J Orthod Dentofac.

[22] Andrews LF (1972). The six keys to normal occlusion. Am J Orthod.

[23] Houston WJ (1983). The analysis of errors in orthodontic measurements. Am J Orthod.

[24] Tamburrino RK. CCO® System: Orthodontic Treatment Design: DENTSPLY GAC International, Inc.; 2018.

[25] Uysal T, Memili B, Usumez S, Sari Z (2005). Dental and Alveolar Arch Widths in Normal Occlusion, class II division 1 and class II division 2. Angle Orthod.

[26] Slaj M, Spalj S, Pavlin D, Illes D, Slaj M (2010). Dental archforms in dentoalveolar Class I. II and III Angle Orthod.

[27] Lee KJ, Jeon HH, Boucher N, Chung CH (2022). Transverse analysis of maxilla and mandible in adults with normal occlusion: a cone beam computed tomography study. J Imaging.

[28] Rastegar-Lari T, Al-Azemi R, Thalib L, Årtun J (2012). Dental arch dimensions of adolescent Kuwaitis with untreated ideal occlusion: variation and validity of proposed expansion indexes. Am J Orthod Dentofacial Orthop.

[29] Cortella S, Shofer FS, Ghafari J (1997). Transverse development of the jaws: norms for the posteroanterior cephalometric analysis. Am J Orthod Dentofac.

[30] McNamara J, Brudon WL, Kokich V (2002). Orthodontics and dentofacial orthopedics. Am J Orthod Dentofac.

[31] Moyers RE (1988). Handbook of orthodontics.

[32] Wagner DM, Chung CH (2005). Transverse growth of the maxilla and mandible in untreated girls with low, average, and high MP-SN angles: a longitudinal study. Am J Orthod Dentofacial Orthop..

